# Age-, season- and gender-specific reference intervals of serum 25-hydroxyvitamin D_3_ for healthy children (0 ~ 18 years old) in Nanning area of China

**DOI:** 10.1186/s12576-023-00895-z

**Published:** 2024-01-02

**Authors:** Dong-yi Zhou, Shang-mou Wei, Chun-ling Zhu, Yu-hong Wei, Xiao-mei Wang, Li-ling Yi, Si-tao Yang, Qi-liu Peng

**Affiliations:** Department of Clinical Laboratory, Guangxi International Zhuang Medicine Hospital Affiliated to Guangxi University of Chinese Medicine, Nanning, 530201 Guangxi China

**Keywords:** Reference intervals (RIs), 25-hydroxyvitamin D_3_ [25(OH)D, Vitamin D, Children

## Abstract

Establishing specific reference intervals (RIs) of serum 25-hydroxyvitamin D3 [25(OH)D] for children is essential for improving the accuracy of diagnosis and prognosis monitoring of diseases such as rickets and growth retardation. The study including 6,627 healthy children was conducted to establish specific RIs of 25(OH)D for children in Nanning area of China. The results showed that there were statistically significant differences among age, season, and gender of serum 25(OH)D levels, and the age-specific RIs of serum 25(OH)D were 20.3 ~ 53.6 ng/mL for 0 ~  ≤ 1 year and 18.9 ~ 49.6 ng/mL for 2 ~  ≤ 3 years. The age-, season-specific RIs of serum 25(OH)D for 4 ~  ≤ 6 years in spring–summer and autumn–winter were 15.8 ~ 42.6 ng/mL and 15.2 ~ 37.7 ng/mL, respectively. The age-, gender-specific RIs of serum 25(OH)D for 7 ~  ≤ 18 years for males and females were 12.1 ~ 36.1 ng/mL and 10.8 ~ 35.3 ng/mL, respectively. This study successfully established the RIs of serum 25(OH)D, which may help to improve disease diagnosis and monitoring for children in the Nanning area of China.

## Introduction

Vitamin D is a fat-soluble steroid vitamin that is essential for the absorption of calcium from the gut and its deposition in the bones [1]. Deficiency of vitamin D in children is associated with a multitude of health problems, including skeletal dysplasias, autoimmune diseases, and neurological disorders [2, 3]. 25-hydroxyvitamin D_3_ [25(OH)D] is the primary circulating form of vitamin D with a relatively high blood content and half-life of 2–3 weeks, which is the most reliable biological marker to assess vitamin D levels [4]. According to previous studies, a variety of elements can influence the 25(OH)D level, such as dietary habits, exposure to sunlight, demographic information (e.g., age and gender), and geographical elements (e.g., season and latitude) [5, 6]. Appropriate reference intervals (RIs) of serum 25(OH)D for children would provide more accurate evidence for the clinical diagnosis and prognostic monitoring of diseases, such as rickets, growth retardation, muscle weakness, and periodontitis. However, there is a lack of appropriate 25(OH)D RIs for children currently, and numerous clinical laboratories still apply RIs determined from adults or from other countries' populations. Accordingly, this study was performed to investigate the influence of age, season, and gender on 25(OH)D levels, and established specific RIs of 25(OH)D for children in Nanning, China.

## Materials and methods

### Participants and recruitment

Referring to the Clinical and Laboratory Standards Institution (CLSI) [7], the research project recruited participants aged 0–18 years old from the Nanning area of China who had received regular physical examinations, nutritional surveillance, and growth monitoring at the Guangxi International Zhuang Medicine Hospital from August 2018 to May 2021. Data for the study was collected by indirect sampling methods to gather information from the hospital's Laboratory Information System (LIS). The criteria for exclusion from the study were as follows: (1) individuals with metabolic diseases, including rickets, obesity, thyroid disease, and Cushing's syndrome; (2) those with recent acute or chronic illnesses related to the heart, liver, lung, kidney, or musculoskeletal system; (3) a family history of diabetes, malignant tumors, parathyroid, and calcium-regulated diseases; (4) those who had recently undergone surgery; (5) those who had recently taken medications that affect the absorption and metabolism of vitamin D, such as vitamin D supplements, glucocorticoids, anticonvulsants, and HAART (AIDS Treatment); (6) laboratory test results that were out of standard ranges, including body mass index (BMI) (0 ~  ≤ 1 year: 14.0–18.0 kg/m^2^; 2 ~  ≤ 3 years: 13.0–18.5 kg/m^2^; 4 ~  ≤ 6 years: 14.0–18.5 kg/m^2^; 7 ~  ≤ 18 years: 14.0–23.8 kg/m^2^), serum parathyroid hormone (PTH) (15–68.3 pg/mL), serum ionized calcium (Ca^2+^) (2.15–2.55 mmol/L), serum thyroid stimulating hormone (TSH) (0.35–4.94 µIU/mL), and serum procalcitonin (PCT) (0–0.5 µg/L). Outliers were removed using Tukey's formula [Q1—1.5 × IQR, Q3 + 1.5 × IQR]. A total of 6,627 apparently healthy individuals were included in the study, with 4,979 being employed to establish RIs and 1,648 being utilized for validating RIs. The healthy individuals were categorized into four age groups: 0 ~  ≤ 1 year (infancy); 2 ~  ≤ 3 years (toddlers); 4 ~  ≤ 6 years (preschool age); and 7 ~  ≤ 18 years (adolescence). Additionally, it was divided into two gender groups: male and female, and four season groups: spring (March ~ May), summer (June ~ August), autumn (September ~ November), and winter (December ~ February). The research protocol was approved by the Guangxi International Zhuang Medicine Hospital (No. 2022-047-01).

### Specimen collection and quality control

Participants were required to fast for 8 h and avoid vigorous exercise for 24 h prior to the collection of a blood sample. Venous blood (2–4 mL) was collected in coagulation-promoting tubes and then centrifuged at 3,000 revolutions per minute for 10 min. The separation of the serum was tested on the Abbott ARCHITECT i2000SR system by the Chemiluminescent Microparticle Immunoassay (CMIA) method within 2 h. Additionally, to ensure the accuracy of the results, Internal Quality Control (IQC) and Performance Qualification (PQ) tests were conducted in compliance with ISO 15189 standards. The system's calibration, linearity, and precision were evaluated by the reference method. Aligning all the tests' analytical performance results with the manufacturer's requirements, the next operation can be commenced.

### Establishment and verification of the RIs

According to the CLSI, the groups with statistical significance were separated, while the groups with no statistical significance were incorporated into a group to establish the RIs. The non-parametrical calculation of percentiles 2.5th and 97.5th were used to calculate the RIs with sample size ≥ 120.

A validation group of 1,648 healthy children was used to validate the accuracy of the established RIs. The validation individuals were categorized into groups according to age, season, and gender-specific RIs, with a sample size of at least 20 in each group. If the validation values are within the range of the RIs for more than 90%, the RIs can be deemed valid. Otherwise, another 20 validation individuals need to be chosen to reassess the results.

### Statistical analysis

The R 4.2.2, IBM SPSS 26.0, and Microsoft Office Excel 2019 Softwares were used for statistical analysis, and all statistical processes were conducted in accordance with CLSI C28-A3 guidelines [7]. The data distribution was described using the Kolmogorov–Smirnov test, and outliers were calculated with Tukey's formula [Q1-1.5 × IQR, Q3 + 1.5 × IQR]. For the non-normally distributed data, medians with quartiles were presented. Spearman's correlation analysis was use to assess the relationship between the 25(OH)D level and age. Nonparametric data were compared by the "ggpubr" R package (version 4.2.1) using the Wilcoxon test and visualization by a violin plot. Bonferroni adjustment was applied for multiple significance tests, and statistical significance was determined by a *P*-value of less than 0.05. RIs were calculated by the nonparametric approach based on the 2.5th and 97.5percentiles. The validation groups were used to verify the established RIs and a passing rate of greater than 90% indicated the RIs were effective.

## Results

### Characteristics of the study subjects

**Fig. 1:**
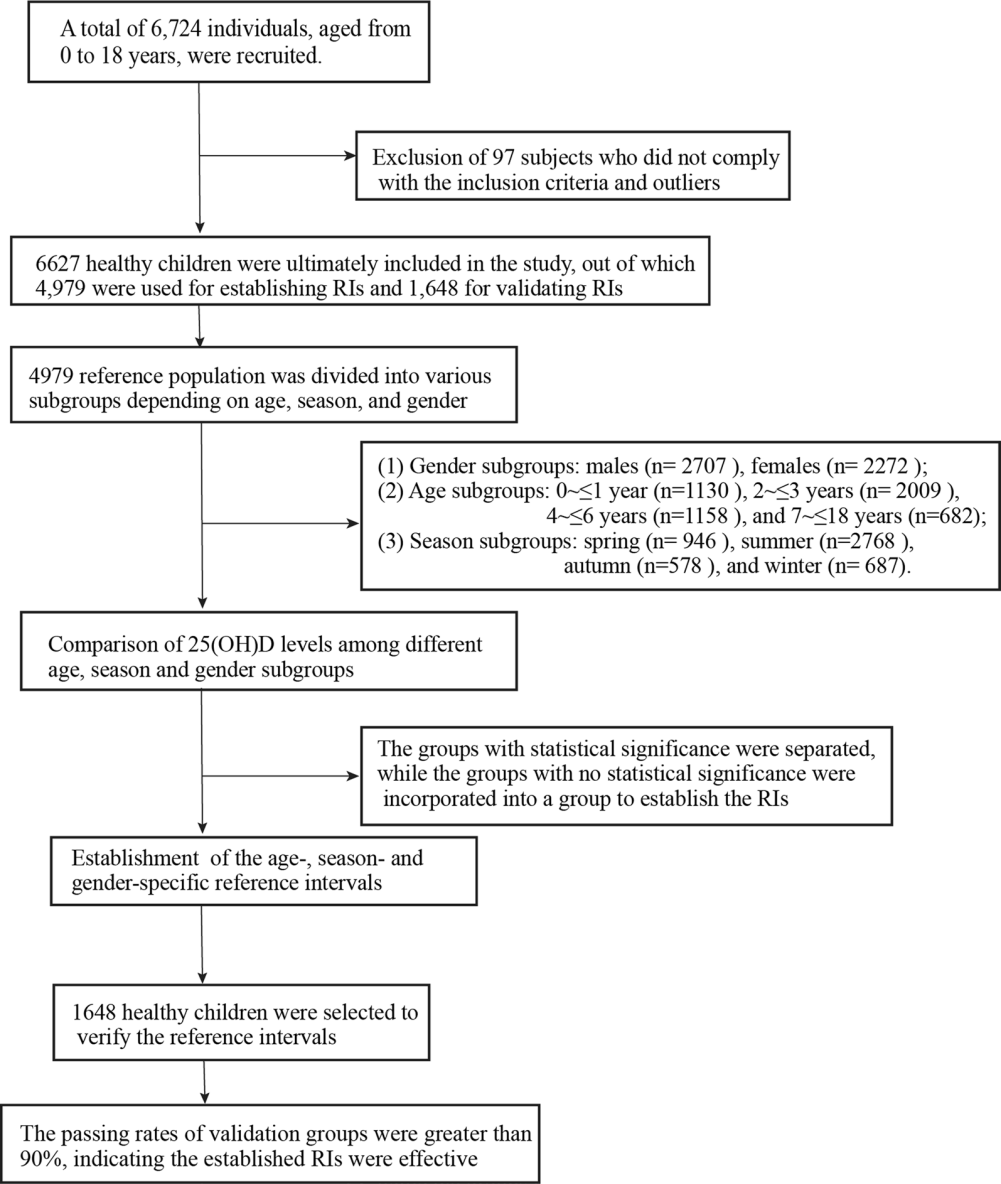
Flow diagram of establishing RIs for 25(OH)D. RIs reference intervals, 25(OH)D 25-hydroxyvitamin D_3_

After analyzing the clinical records of the participants and removing the outliers, a total of 6,627 healthy children were selected, with 4,979 were used to establish RIs and 1,648 were employed for validating them (Fig. 1). The characteristics of the study subjects were summarized in Table 1.

**Table 1:** Characteristics of study participants (n = 6627)

Parameters	Infancy (0 ~ ≤ 1 year)	Toddlers (2 ~ ≤ 3 years)	Preschool age (4 ~ ≤ 6 years)	Adolescence (7 ~ ≤ 18 years)
Age(years)	0.76 ± 0.30	2.6 ± 0.48	4.6 ± 0.71	9.42 ± 2.3
Gender(M/F)	814/728	1304/1117	869/701	544/550
BMI(kg/m^2^)	15.8 ± 1.6	16.7 ± 2.09	17.8 ± 1.5	21.36 ± 2.6
PTH(pg/mL)	26.11 ± 11.47	28.67 ± 11.28	24.75 ± 9.4	39.9 ± 11
Ca^2+^(mmol/L)	2.37 ± 0.42	2.39 ± 0.08	2.37 ± 0.05	2.4 ± 0.09
TSH(µIU/mL)	1.65 ± 0.93	2.42 ± 1.0	2.18 ± 0.79	2.48 ± 1.24
PCT(µg/L)	0.18 ± 0.05	0.23 ± 0.12	0.22 ± 0.11	0.18 ± 0.07

*BMI* body mass index, *PTH* parathyroid hormone, *Ca*^*2*+^ ionized calcium, *TSH* thyroid stimulating hormone, *PCT* procalcitonin

### Comparison of 25(OH)D levels among different age, season and gender subgroups

**Fig. 2:**
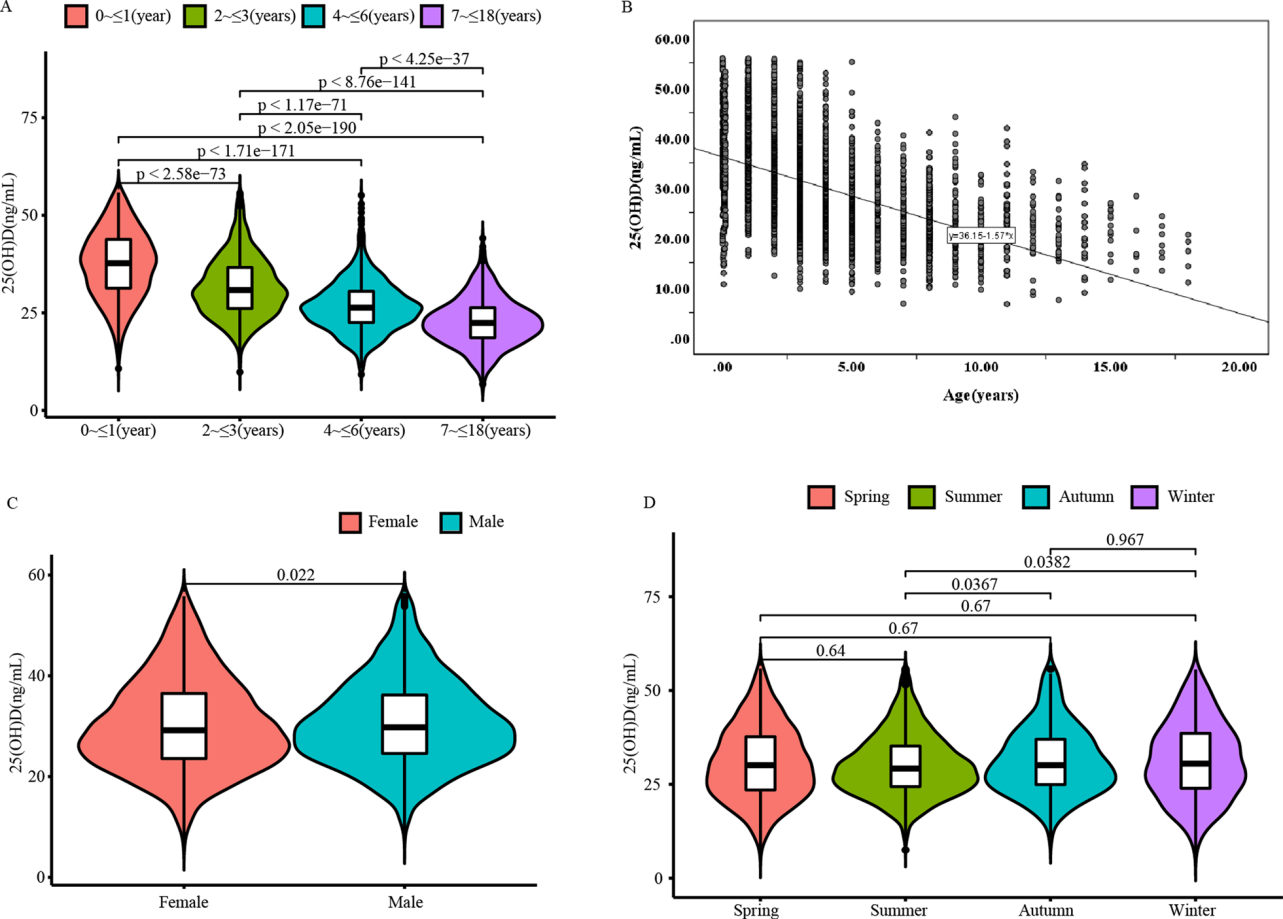
Comparison of 25(OH)D levels among different age, season and gender groups. **A** The vioplot showed the differential expression of 25(OH)D levels among different age groups. **B** Identifying the correlation between 25(OH)D levels and age for healthy children. **C** The vioplot showed the differential expression of 25(OH)D levels between different genders. **D** The vioplot showed the differential expression of 25(OH)D levels among seasons. 25(OH)D 25-hydroxyvitamin D_3_

The data of serum 25(OH)D was skew distribution according to the analysis of the Kolmogorov–Smirnov test (*P* < 0.05), and statistics of 25(OH)D level were presented as medians with quartiles. Results of statistical analysis revealed that 25(OH)D levels declined with age increasing (r = −0.52, *P* < 0.05), showing statistically significant differences in the 0 ~  ≤ 1 year, 2 ~  ≤ 3 years, 4 ~  ≤ 6 years, and 7 ~  ≤ 18 years age groups (all *P* < 0.001, Fig. 2A, B). Additionally, there were differences between males and females in their serum 25(OH)D levels (*P* < 0.05, Fig. 2C). Moreover, the serum 25(OH)D levels in summer were significantly higher than in winter, while lower than in autumn (all *P* < 0.05). There were no statistically significant differences between the serum 25(OH)D levels in autumn and winter, or in spring and summer (all *P* > 0.05, Fig. 2D). Consequently, the autumn and winter groups were incorporated into an autumn–winter group, the spring and summer groups were merged into a spring–summer group.

### Differences of 25(OH)D levels among genders and seasons for the same age groups

**Fig. 3:**
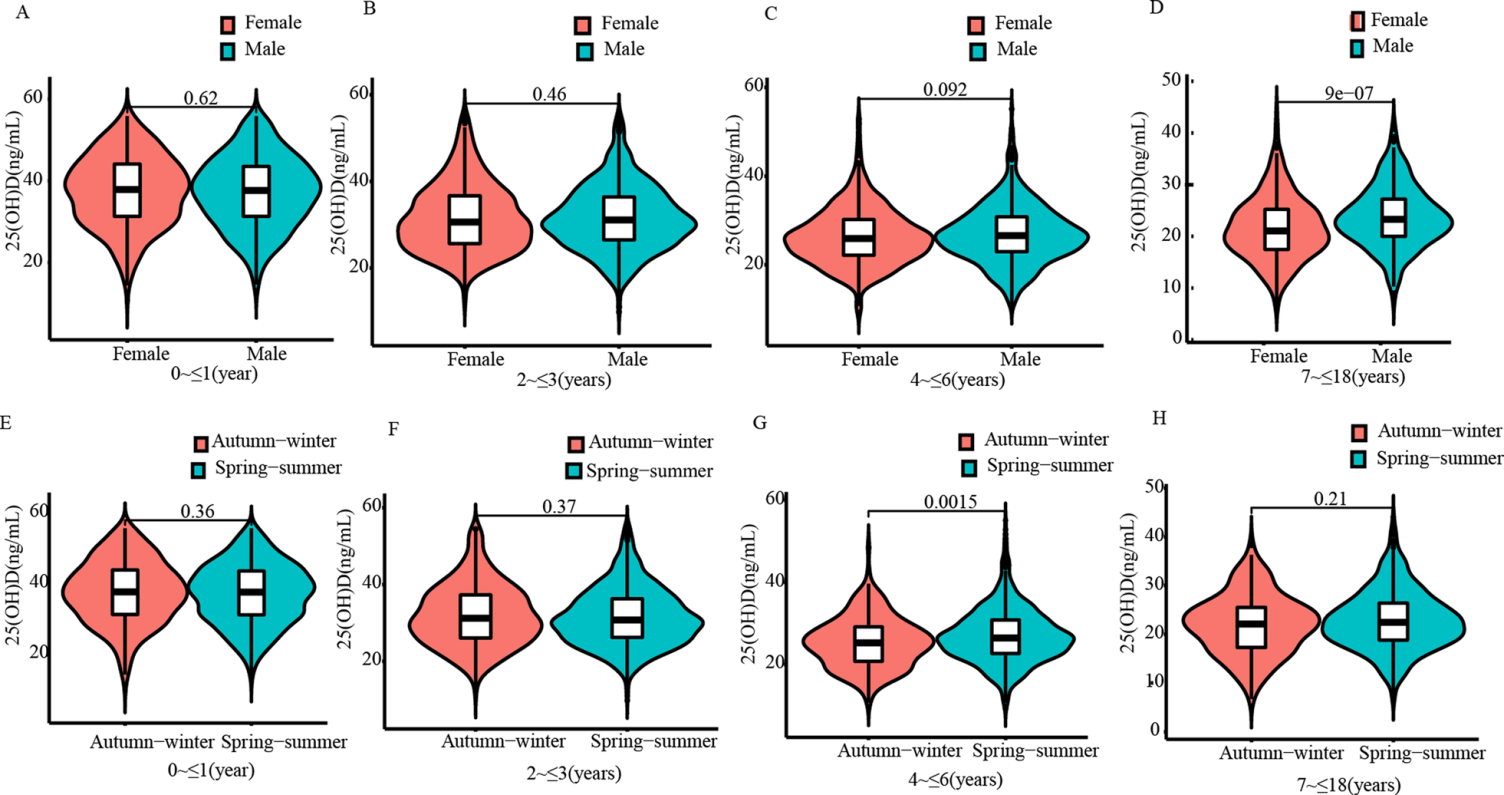
Gender and seasons differences for 25(OH)D levels in different age groups. **A**–**D** The vioplot showed the differential expression of 25(OH)D levels between different genders in different age groups. **E**–**H** The vioplot showed the differential expression of 25(OH)D levels between different seasons in different age groups. 25(OH)D 25-hydroxyvitamin D_3_

Statistical analysis revealed that males aged 7 ~  ≤ 18 years old had significantly higher 25(OH)D levels than females (*P* < 0.05, Fig. 3D). However, there was no significant difference between males and females aged 0 ~  ≤ 6 years (all *P* > 0.05, Fig. 3A–C). Additionally, a significant difference in serum 25(OH)D level was observed between spring–summer and autumn–winter in 4 ~  ≤ 6 years (*P* < 0.05, Fig. 3G). No significant differences were observed between spring–summer and autumn–winter in 0 ~  ≤ 1 year, 2 ~  ≤ 3 years, and 7 ~  ≤ 18 years (all *P* > 0.05, Fig. 3E, F and H). As a result, the RIs among 4- ≤ 6 years were divided by seasons, while those among 7 ~  ≤ 18 years were divided by gender.

### Establishment and verification of the RIs

The RIs were determined by the non-parametrical calculation of percentiles 2.5th and 97.5th. The age-specific RIs of serum 25(OH)D were 20.3 ~ 53.6 ng/mL for 0 ~  ≤ 1 year and 18.9 ~ 49.6 ng/mL for 2 ~  ≤ 3 years. The age-, season-specific RIs of serum 25(OH)D for 4 ~  ≤ 6 years in spring–summer and autumn–winter were 15.8 ~ 42.6 ng/mL and 15.2 ~ 37.7 ng/mL, respectively. The age-, gender-specific RIs of serum 25(OH)D for 7 ~  ≤ 18 years for males and females were 12.1 ~ 36.1 ng/mL and 10.8 ~ 35.3 ng/mL, respectively (Table 2). Subsequently, 1648 healthy subjects were selected to verify the established RIs, with more than 20 cases in each validation group. Finally, it was found that the percentages of passing rate for all validation values were greater than 90%, indicating that the RIs established in this study were effective (Table 2).

**Table 2:** Age-, season- and gender-specific medians and RIs of 25(OH)D for healthy children

Age(years)	Groups	Cases	25(OH)D (ng/mL)	95% CI	*P*	RIs (P2.5 ~ P97.5)	Range of validated values	Passed rate (%)
0 ~ ≤ 1(year)	Male	608	37.6 (31.3 ~ 43.5)	36.6 ~ 37.9	> 0.05	20.3 ~ 53.6	23.4 ~ 51.5	100
	Female	522	37.9 (31.3 ~ 44.1)	36.8 ~ 38.3				
	Spring–summer	705	37.7 (31.3 ~ 43.7)	36.6 ~ 37.8	> 0.05			
	Autumn–winter	425	37.9 (31.3 ~ 44.1)	36.8 ~ 38.5				
2 ~ ≤ 3(years)	Male	1098	31.1 (26.5 ~ 36.4)	31.2 ~ 32.1	> 0.05	18.9 ~ 49.6	17.9 ~ 50.1	92
	Female	911	30.6 (25.7 ~ 36.7)	31.2 ~ 32.2				
	Spring–summer	1574	30.7 (26.2 ~ 36.3)	31.2 ~ 31.9	> 0.05			
	Autumn–winter	435	30.6 (25.7 ~ 36.7)	31.3 ~ 32.8				
4 ~ ≤ 6(years)	Male	663	26.6 (22.9 ~ 30.8)	26.4 ~ 27.4	> 0.05	Spring–summer: 15.8 ~ 42.6	16.9 ~ 40.1	100
	Female	495	25.9 (22.1 ~ 30.2)	25.8 ~ 26.9		Autumn–winter: 15.2 ~ 37.7	15.9 ~ 41.2	98
	Spring–summer	925	26.5 (22.7 ~ 30.9)	26.6 ~ 27.4	< 0.05			
	Autumn–winter	233	25.9 (22.1 ~ 30.2)	24.6 ~ 26.2				
7 ~ ≤ 18(years)	Male	338	23.3 (20.0 ~ 27.2)	23.2 ~ 24.4	0.001	Male: 12.1 ~ 36.1	12.0 ~ 35.5	97
	Female	344	21.1 (17.5 ~ 25.3)	20.9 ~ 22.3		Female: 10.8 ~ 35.3	13.1 ~ 35.4	97
	Spring–summer	510	23.4 (18.8 ~ 26.4)	22.4 ~ 23.5	> 0.05			
	Autumn–winter	172	21.1 (17.5 ~ 25.3)	21.1 ~ 22.9				

*IQR* interquartile ranges, *CI* Confidence Interval, *RIs* reference intervals, *25(OH)D* 25-hydroxyvitamin D_3_

## Discussion

Vitamin D deficiency has become a global health issue, especially in children [8]. Statistics from a 2017 Chinese Epidemiological Survey show that 23.2% of Chinese children suffer from Vitamin D deficiency [9]. A study conducted in England between 2008 and 2014 revealed a 15- fold rise in the number of young people with vitamin D insufficiency [10]. Similarly, a 2018 study in India showed that 41.9% of children and adolescents had inadequate levels of vitamin D [11]. Vitamin D deficiency can lead to poor bone formation and a higher risk of fractures [12]. Additionally, research has suggested that insufficient levels of vitamin D may be connected to cardiovascular disease, various kinds of cancer, and diabetes [13–16]. Early diagnosis and appropriate vitamin D supplementation can reduce the prevalence of the disease. In this research, we established gender-, age-, and season-specific RIs of serum 25(OH)D for children, providing more accurate evidence for diagnosing and treating the disease.

Our findings indicated that there were statistically significant variations in the 0 ~  ≤ 1 year, 2 ~  ≤ 3 years, 4 ~  ≤ 6 years, and 7 ~  ≤ 18 years age groups (all *P* < 0.001), and the serum 25(OH)D levels decreased with age increasing, which is in agreement with prior studies [17–19]. The trend may be attributed to the young children's daily intake of vitamin D-rich infant formula as well as fortified baby foods, while adolescents' lack of outdoor activities and inadequate vitamin D supplementation. In addition, adolescents experience a period of accelerated growth, which necessitates a considerable amount of vitamin D for the development of healthy musculoskeletal and neurological systems. Another important finding was that the level of serum 25(OH)D in males was significantly higher than in females aged 7 ~  ≤ 18 years (*P* < 0.05), which is consistent with the European children, southern Chinese and Iranian children [19, 20]. This can be attributed to the male hormone levels, as testosterone is believed to have a positive correlation with the levels of 25(OH)D [21, 22]. A different study, however, suggested that there was no significant distinction based on sex, race, or age [23]. These variations could be associated with lifestyle elements, such as dietary habits and outdoor activities of the studied population. It was discovered that the serum 25(OH)D levels during summer were significantly lower compared to autumn, while higher than in winter (all* P* < 0.001). The findings for the seasonal fluctuation in serum 25(OH) levels were close to those from other research projects carried out in the Zigong area and southern China [17, 20]. The varying levels of 25(OH)D can be explained by the amount of sunlight exposure, since it is mainly acquired through exposure to the sun's UV rays that activate its production in the skin. Compared to the autumn–winter groups, the 25(OH)D concentration for children aged 4 ~  ≤ 6 years was higher in the spring–summer groups, which could be attributed to the lack of light exposure and outdoor activities in the cold winter months.

This study investigated the age-, season- and gender-specific RIs of serum 25(OH)D for healthy children (0–18 years old) in the Nanning area, which is located in 22.5° north latitude. The levels of serum 25(OH)D in Nanning (22.5° north) were found to be higher than those in Shanghai (30.5° north) and Sichuan (27.5° north), which could be attributed to the difference in geographical latitudes [24, 25]. As the reports suggest, the further away from the equator, the lower the concentrations of 20(OH)D and the higher the prevalence of Vitamin D insufficiency presenting in children due to the decreased amount of sunlight [26]**.** In addition, the higher serum 25(OH)D levels in our study may be explained by the differences in detection methods, as the metabolites in the serum will increase cross-reactivity with the antibodies in the CMIA, whereas the liquid chromatography tandem mass spectrometry (LC–MS/MS) method is less affected by the metabolites. Moreover, the serum 25(OH)D RIs in this research were more precise and different from the reagent manufacturer's recommended levels (≥ 20 ng/mL), which can be attributed to the age range, ethnic group, and geographical area of the sample population, as the sample of the reagent manufacturer comprises of African-American, Hispanic, and Caucasian adults from the 48 contiguous states of the U.S. In the validation test, the passing rates for all validation groups were greater than 90%, indicating that the established RIs in the current study were effective, which will be more accurate and significant for clinical diagnosis of diseases in children. However, one major limitation of this research is that there are no specific RIs of 25(OH)D for other special populations, such as elderly and pregnant women. Therefore, further researches based on a wider range of people should be performed to establish more comprehensive RIs.

## Data Availability

The dataset generated and/or analyzed during this study are available from the corresponding author on reasonable request.
